# The effect and cost-effectiveness of a group-based parenting intervention for parents of preschool children with subclinical neurodevelopmental disorders and mental health problems: protocol for a multiple-baseline single-case experimental design (SCED) with a pre-, post and follow-up

**DOI:** 10.1186/s40359-025-02618-y

**Published:** 2025-03-27

**Authors:** Anton Dahlberg, Vedrana Bolic Baric, Filipa Sampaio, Karin Fängström

**Affiliations:** 1 Child Health and Parenting (CHAP), Department of Public Health and Caring Sciences, Uppsala University, Uppsala, Sweden; 2 Department of Health, Medicine and Caring Sciences, Linköping University, Linköping, Sweden; 3 Akademiska Children’s Hospital, Uppsala University Hospital, Uppsala, Sweden

**Keywords:** Young children, Subclinical neurodevelopmental disorders, Emotional and behavioral problems, Parenting support

## Abstract

**Background:**

Young children with subclinical neurodevelopmental disorders (NDDs) and concurrent emotional and behavioral problems (EBP) are at significant risk of negative short- and long-term outcomes. Although early parenting support interventions are recommended and requested, there is a lack of interventions specifically designed for this group and adapted to the Swedish context. Based on this gap, a parenting support intervention for parents with children aged 2–6 years with subclinical NDDs and EBP has been co-created with clinicians and parents. The project described in this study protocol aims to evaluate the effectiveness and cost-effectiveness of this new group-based parenting intervention.

**Methods:**

The project uses a multiple-baseline single-case experimental design (SCED) with pre-post measures and a 3-month follow-up. The intervention is provided to families with children who are referred to child health psychologists at the child pediatric outpatient clinic in Uppsala Region, Sweden. Outcomes will include child EBP and parent self-efficacy, stress, well-being, and quality of life, as well as costs for the intervention, health care use, and QALYs.

**Discussion:**

The project could lead to improved mental health in both children and parents through participation in the group-based parenting intervention. The study design, with longitudinal data from both children and/or their parents, will provide valuable insights into the trajectories of mental health and well-being within this group. In addition, the inclusion of young children as informants will provide important information about their experiences. Furthermore, the use of pre-, post- and follow-up questionnaires will allow reliable and clinically significant changes to be assessed and our findings to be compared with randomized trials in similar populations. The results of this project will be relevant to children with subclinical NDDs and their parents, as well as to health care organizations and the scientific community. The intervention is well adapted to the end users and the clinical context, as it has been co-created with clinicians and parents.

**Trial registration:**

ISRCTN10835479 10.1186/ISRCTN10835479, date of registration 2024-10-08.

## Background

There is a large body of research on parenting interventions as a key strategy for improving early child development outcomes for children diagnosed with neurodevelopmental disorders (NDDs) [1]. NDDs are characterized by symptoms that emerge in early childhood and include the diagnoses of autism spectrum disorders (ASD), attention deficit hyperactivity disorder (ADHD), developmental language disorders, learning disabilities, and motor disorders [2]. NDDs affect approximately 10–15% of children, and they have a significant impact on behavior, well-being and everyday functioning [3]. However, the comorbidity between different developmental disabilities tends to be high [4, 5], which in turn may result in poorer outcomes than isolated challenges [6].

Subclinical NDDs, also sometimes referred to as sub-threshold cases of NDDs, is a broader term that includes children who have insufficient symptoms to make a diagnosis but are impaired [7]. Compared to children with a diagnosed NDD, children with subclinical NDDs are an understudied group, often presenting with extensive difficulties and problems [8] but without having access to the support and care provided to families of children with a diagnosis.

Children with NDDs are more likely to have mental health problems compared to their peers [9, 10]. Mental health problems in turn are globally one of the main causes of burden of disease among children [11], affecting approximately 10–20% of all children [12]. In preschool children mental health problems are displayed as emotional and behavioral problems (EBP), and the prevalence is about 7–18% [6]. The double exposure of both subclinical NDDs and EBP may further increase the risk of short- and long-term negative consequences. Indeed, studies have shown that subclinical NDDs during childhood predict poorer functional outcomes and increased mental health problems in adults [7].

The health and well-being of parents of children with a diagnosed NDD is well studied [13–15]. These parents often have heightened parental stress [8, 16], lower well-being and poorer self-efficacy compared to parents of typically developing children [17]. Less research has been conducted on parents of children with subclinical NDDs. However, existing research indicates that these parents may also experience impaired health and well-being [18, 19].

Early interventions targeting children with subclinical NDDs and EBP are both recommended and wanted [20–24]. Parenting interventions have been shown to be among the most effective early interventions for improving children’s mental health and reversing negative child outcomes There are many evidence-based parenting programs targeting typically developing children and the vast number of scientific studies of these programs have made it possible to investigate the components that are most effective in reducing child EBP [24]. However, with a few exceptions, these standard programs do not entail specific strategies or approaches designed for and adapted to children with a disability [25]. Interventions targeting parents of children with disabilities often utilize applied behavior analysis (ABA) to address specific behavior or skills training to decrease maladaptive behavior and improve communication and socialization [26]. One important focus is on antecedent strategies which are used to create conditions that prevent the problem behavior before it occurs [26, 27]. Focusing on adapting and adjusting the environment and strategies to enhance the child’s functionality in everyday life in order to improve outcomes also aligns with the World Health Organization’s International Classification of Functioning, Disability and Health (ICF) framework [28]. The ICF proposes a biopsychosocial framework that stresses the interaction between personal, social and environmental factors to find supportive solutions with the aim of improving well-being and quality of life [29]. In addition to specific strategies taught, the effect of all parenting group interventions also depends on parents completing programs [30]. This in turn is influenced by the group itself and parents stress the importance of meeting other parents with similar experiences to have a sense of belonging, to share experiences and feel safe talking and to support each other [21, 30, 31]. A qualitative interview study with parents of children with subclinical NDDs demonstrated that these parents longed to meet other families with similar difficulties [21].

The perspectives and experiences of young children are seldom included in research on parenting programs, nor on children with NDDs and EBP. This is despite the fact that Sweden ratified the UN Convention on the Rights of the Child in 1990 and since 2020 it has been part of Swedish law. It is crucial to consider children’s perspectives to gain insight into their daily family lives and the impact of parenting programs.

In Sweden, parenting support programs are either provided as a universal intervention by the municipalities (e.g., Cope, Triple P, or Connect) or by the child psychiatric clinics or the Habilitation services to families where children have a diagnosed disorder (e.g., Komet, Strategi, or individual therapy based on applied behavior analysis). There is thus a lack of early interventions specifically designed to parents of preschool aged children with subclinical NDDs adapted to a Swedish context.

This study is based on a quality improvement initiative to enhance the healthcare process for this target group in the Uppsala Region, Sweden. The work resulted in the development of a new care model and one of the key aspects of this model is a new group-based parenting intervention called Everyday Life and Parenting, which is described in detail in the Methods section. The aim of this study is to evaluate the effects and cost-effectiveness of the group-based parenting intervention Everyday Life and Parenting. Further, the project aims to explore trajectories of mental health and well-being of children and parents over time.

## Objectives

### Primary objectives

Objective 1: To evaluate the impact of the Everyday Life and Parenting intervention on child behavior problems.

Objective 2: To evaluate the impact of the Everyday Life and Parenting intervention on parental self-efficacy and parental stress.

### Secondary objectives

Objective 3: To assess changes in mental health problems and quality of life before and after participating in the Everyday Life and Parenting intervention.

Objective 4: To undertake a cost-effectiveness analysis.

Objective 5: To explore the trajectories of children’s and parents’ mental health and wellbeing over time.

## Methods

### Design

This study employs a prospective, longitudinal design, utilizing repeated assessments to observe changes in key outcome measures from pre- to post-intervention, with follow-up assessments conducted to evaluate the sustained effects over time. The study will run from August 2024 to December 2025. It combines two distinct yet complementary approaches. Firstly, a multiple baseline single case experimental design (SCED) is employed across participants, which permits a detailed analysis of individual responses to the intervention over time and a nuanced understanding of behavioral change at the participant level. This design is useful when testing the effect of an intervention in a heterogenous group of patients or when examining a known intervention for patients other than those it was originally designed for [32]. Secondly, a traditional pre-, post- and follow-up assessment using standardized questionnaires is conducted to capture broader intervention effects across the participant group, evaluating changes in key outcome measures over time. Collectively, these methods provide both detailed case-specific insights and generalizable findings.

### Participants

The target population is children aged 2–6 years with subclinical levels of NDDs and behavioral problems and their parents. The children have been referred to child health psychologists at the child pediatric outpatient clinic in Uppsala Region and offered the parenting group intervention Everyday Life and Parenting. To be eligible for the project, the parents must attend the intervention and be able to fill in questionnaires and answer weekly measurements in Swedish. Children aged 4–6 years whose parents consent to participate in the project will be invited to participate. No other inclusion or exclusion criteria will be applied.

According to the SCED standard, at least three, preferably five, participants should be included in each baseline condition [32]. The goal in our study is to retain 15 parents and 15 children as participants, i.e., 5 parents and 5 children in each of the three baseline conditions. To take into account an expected drop-out of about 10–20% we will need to recruit about 18 parents and 18 children [33]. This is deemed to be more than sufficient to be able to make detailed SCED analyses.

In terms of pre-, post- and follow-up assessment, we want to be able to detect relatively small effect sizes. To be able to detect an effect size of 0.35 with an alpha of 0.05 and power of 80%, we will need at least 54 participating parents. If we assume a 20% attrition rate, 70 participants will need to be recruited. These results can be compared to randomized studies on similar populations.

### Recruitment

Information about the research project (aimed at parent and child respectively) will be sent home to families offered the intervention, along with information about the parenting intervention. Staff from the child pediatric outpatient clinic will call the family to provide information about the intervention and ask the parent if they consent to participate in the study. If they agree, informed consents are documented by the staff in a secure online platform called Research Electronic Data Capture (REDCap). REDCap is specifically geared to support online and offline data capture for research studies and operations. Researchers will have access to participants’ consents and contact details via REDCap.

Only one parent per family can participate in the research project. We will endeavor to achieve an even distribution of mothers and fathers as research subjects. In cases where both parents in a family wish to participate in the research, the parent who favors the aim of gender balance will be selected.

Children aged 4–6 years, whose parent has been asked and agreed to participate in the project, will be given the opportunity to participate themselves. Specific information about the study adapted for children will be sent home together with the study information for parents. The parent is asked to read this information to their child. If the child has two guardians, the consent of both is needed for the child to participate in the study.

Recruitment and data collection started in August 2024 and will continue throughout 2025.

### The group-based parenting intervention Everyday Life and Parenting

The Everyday Life and Parenting program targets parents of children aged 2–6 years with subclinical levels of NDDs and behavioral problems. These children are referred by child psychologists at the Uppsala Region’s child pediatric outpatient clinic. Families access the intervention through routine care triage, either from the waiting list or via recommendation by their consulting psychologist.

The Everyday Life and Parenting intervention consists of four modules. The intervention is theoretically based on social-learning theory [34] including ABA [35], the ICF framework and theory on emotion regulation [36, 37].

Three of the intervention modules include key components from the most effective parenting programs targeting behavior problems in children [24], including positive reinforcement techniques, relationship enhancement techniques and reframing unhelpful cognitive perceptions about the child, as well as emotion regulation knowledge and techniques. The fourth module is based on ABA and teaches a range of antecedent strategies [26, 35], such as how to organize the physical environment, how to use visual aids such as schemes of daily routines and time timers, and how to use high-probability request techniques. Furthermore, the modules include teaching parents general analytical and learning principles for teaching new skills or dealing with problem behaviors. In addition, parents are taught how to adapt activities and demands to the individual child, based both on his/her strengths, and on cognitive and adaptive abilities. Parents also practice reflecting on their own emotions, cognitions and abilities in order to find ways of adjusting and adapting to improve their own and their child’s well-being. This holistic approach to the child’s and the parents’ functioning is in line with the ICF framework. The delivery format of the intervention is also based on well researched components [38] and includes providing psychoeducation; discussions; formulating goals and goal attainment; homework assignments and peer support. Each module lasts 3 h and the intervention is given every other week for 8 weeks.

Depending on the parents’ preferences, the intervention is delivered either entirely on-site or digitally. In the digital option, the first session is on-site to allow parents to get to know each other. The other sessions are digital.

Groups of Everyday Life and Parenting start four to six times every term. Each group includes parents from up to 10 families, i.e., in total 20 parents. Both parents are encouraged to participate in the intervention.

### Materials

Primary outcomes are assessed through the parent SCED data collection, while the secondary outcomes are assessed through child SCEDs, pre/post-measures and the child’s medical records.

Primary outcomes.

- Child behavior problems.
- Parental self-efficacy.
- Parental stress.

Secondary outcomes.

- Child mental health problems.
- Parents’ mental health.
- Parents’ quality of life.
- Goal attainment and client satisfaction.
- Child medical data.
- Children’s positive and negative experiences of family life.

In the current study, data are collected from five sources:

- Parent weekly short measures.
- Validated questionnaires at pre-, post- and 3-month follow-up (T1, T2, T3).
- Forms on goal, goal attainment and client satisfaction with the intervention.
- Data from the child’s medical records.
- Child weekly short measures.

#### Parent weekly short measures

The short measure that parents will answer weekly was developed specifically for this project. It contains 25 questions on children’s behaviors, on parents’ self-efficacy and on parents’ perceived stress. The questions on children’s behaviors are based on the validated Eyberg Child Behavior Inventory [39], the questions on self-efficacy are from the validated Me As a Parent questionnaire [40] and the questions on stress are from the Parental Stress Scale [41]. The questions were modified together with the project’s Parent Council (parents who had previously participated in the Everyday Life and Parenting) as well as with the Clinical expert group (consisting of a child psychologist and a special education teacher who delivers the intervention) to fit the context. Basing the questions on validated forms increases the reliability of the results.

#### Demographic data

Background information will be collected at T1. Questions on the parent: age; sex; level of education; country of birth and number of years in Sweden; language/s spoken at home; economic worries; main occupation. Questions on the child: sex; age; number of siblings; age of siblings; whether any of the children have a diagnosed neuropsychiatric disorder (ADHD, ASD, language disorder, etc.). In addition, it is asked whether anyone else in the family, such as another parent, is participating in the intervention.

#### Pre-, post- and 3-month follow-up

**Child behavior problems.** Child behavior problems will be assessed using the Eyberg Child Behavior Inventory (ECBI) [42]. The ECBI is a 36-item rating scale measuring disruptive behavior problems in children and it assesses both frequency of a behavior and whether the parent perceives the behavior as a problem. The Swedish version of ECBI has good psychometric properties and is sensitive to change [39].

**Child mental health problems**. In addition to child behavior problems, we will also assess child emotional problems, peer problems and prosocial functioning, using the Strengths and Difficulties Questionnaire (SDQ). Thus, the SDQ capture the child’s mental health problems, and it has been validated in a non-clinical sample of children aged 3–5 years old in Sweden [43, 44].

**Parental self-efficacy.** Me As a Parent (MaaP) will be used to measure parents’ ratings of their own parenting skills and parental self-efficacy. It contains 16 questions measuring confidence in their own abilities, sense of control as a parent, ability to handle situations with children and self-direction [40].

**Parental stress.** The Parental Stress Scale (PSS) will be used to measure parental stress. It consists of 18 questions in two dimensions - satisfaction with parenting and stress [41]. We will only use the questions covering stress.

**Parents’ mental health.** The General Health Questionnaire (GHQ-12) will be used to measure parental mental health. The GHQ-12 measures symptoms of mental health problems, such as depression and anxiety, and participation in everyday social contexts. The GHQ-12 is a validated and well-used instrument in research and evaluation, both globally and in Sweden [45].

**Parents´ quality of life.** Quality of life among parents will be measured using the Assessing Quality of Life 8 Dimensions (AQOL-8D), which measures quality of life in adults for use in economic evaluation, based on eight dimensions, including independent living, mental health, coping, relationships, pain, self-worth, happiness and senses) and 35 items with four to six response levels, each representing increasing levels of severity [46].

**Resource use.** Costs related to the consumption of societal resources will be collected using an adapted version of the Trimbos/iMTA questionnaire for costs associated with Psychiatric Illness (TIC-P). It comprises information on the use of resources related to healthcare, medication and supplements, social support and assistance, and productivity losses due to absence from paid and unpaid work for parents for care of a sick child [47].

#### Goal, goal attainment and client satisfaction

At the beginning of the group intervention the participants set up goals for themselves and their family. At the end of the intervention, they evaluate goal attainment as well as their satisfaction with the intervention. This data will be used to understand more about implementation aspects of the intervention [48].

#### Child medical records

The following health data will be collected: number of healthcare visits (e.g. doctor, nurse, speech therapist, psychologist, dietician, physiotherapist), visits to the emergency department, inpatient care, prescribed drugs, referrals, child development with regard to symptoms of NDDs.

#### Child weekly short measures

The short measure of 5 questions uses visual aids and a simple rating scale. It was developed based on the researchers’ previous studies with preschool-aged children [49–51] and questions used in previous studies with children whose parents participate in a parenting intervention [52]. The questions have been reviewed by the project’s Parent Council and revised accordingly. They cover both children’s positive and negative experiences in the family. The questions will be recorded and read out loud. This increases the possibility for children to answer without adult interference, which can increase reliability.

### Procedure

Parent weekly measures will be collected via telephone by research assistants, with responses entered into REDCap. Data collection spans 15, 18, or 21 weeks, depending on the participant’s baseline length (4, 7, or 10 weeks). Pre-, post-, and 3-month follow-up questionnaires will also be distributed via REDCap, with pre-measures sent before baseline and post-measures after the intervention. Goal setting, goal attainment, and client satisfaction forms will be sourced from the Everyday Life and Parenting materials.

Child health data will be obtained through the Uppsala Region’s procedure for accessing research data from the electronic medical record system Cosmic. Child weekly measures will be sent digitally via REDCap and completed during the same weeks as the parent measures.

Intervention fidelity will be assessed through a checklist completed by course leaders after each module, indicating which session elements were completed.

### Analysis

The data will be analyzed using SCED methods and linear mixed-effects models to capture both individual-level changes and group-level effects over time.

### SCED analysis

The items from the weekly measurements will be displayed in graphs and inspected visually, which is standard practice in SCED [32]. The graphs will be evaluated regarding direction and slope of change in the data, i.e., the trend, as well as onset, that is, when change occurs. This will be done separately for each participant.

When using SCED it is recommended to supplement the visual analysis with calculating the effect size in order to provide standardized and reliable indices. We will use Nonoverlap of All Pairs (NAP) in which each data point in Phase B (intervention) is compared with each data point in Phase A (baseline) [53]. NAP effect sizes of 0-0.65, 0.66–0.92, and 0.93-1.0 corresponded to small, moderate, and large effects based on expert visual judgment [53].

### Reliable change

To obtain a picture of whether changes in intensity and frequency of child behavior problems (ECBI) are both reliable and clinically significant, we will classify children as recovered, improved, unchanged or deteriorated. This will be done by incorporating both a measure of whether the change in scores is larger than what is expected due to measurement error of the tool i.e., statistical significance (Reliable Change Index - RCI) as well as the participant’s shift from a clinical state to a non-clinical state i.e., clinical significance (Clinically Significant Change– CSC) [54]. This is calculated for each individual based on pre- and post-data, and presented as percentages (percentage of children who were classified as recovered, improved, unchanged and deteriorated). We will also conduct sub-group analysis comparing girls and boys. Even though the sample will be too small for detecting small to medium effects in sub-group analyses, including such analyses will enable meta-analyses to do so.

### Mixed-effects models analysis

For the data collected at pre-, post-, and 3-month follow-up using standardized questionnaires, linear mixed-effects models will be employed to analyze changes over time in key outcome measures. These measures include child behavior problems (ECBI), child mental health problems (SDQ), parental self-efficacy (MaaP), parental stress (PSS), and parents’ mental health (GHQ-12). Mixed-effects models are appropriate for repeated measures data as they account for within-subject correlations and can handle missing data. This approach allows for the inclusion of all available data, maximizing statistical power even if some participants have missing data at one time point [55, 56].

### Health economic evaluation

The health economic evaluation will be performed according to the Consolidated Health Economic Evaluation Reporting Standards 2022 Statement [57] and will include the following analyses: (1) a cost-effectiveness analysis using cost per improved case (based on the RCI/CSC) as the outcome, and (2) a series of cost-utility analyses using cost per quality adjusted life years (QALYs) gained for children (using SDQ scores mapped onto a multi-attribute utility instrument using available algorithms), for parents (using the scores from the AQOL-8D) and for the dyad as the outcome.

Costs will be analyzed from two different perspectives: a health care provider perspective and a societal perspective. The health care provider perspective includes the cost to run the intervention (e.g., therapist salary costs, administration) which will be collected from project documentation at the clinic. The societal perspective includes, in addition to intervention costs, other health care costs (health care utilization and medication), as well as costs beyond health care, including social support, and indirect costs related to lost productivity for parents (related to absence from paid and unpaid work) collected via the TIC-P.

Total costs and total QALYs will be aggregated over the trial period and estimated using the area under the curve method [58]. Differences in improved cases, total QALYs and total costs for children and parents before and after the intervention will be analyzed using regression models.

In the cost-effectiveness and cost-utility analyses, we will estimate an incremental cost-effectiveness ratio (ICER) as the ratio between the difference in costs and the difference in improved cases and QALYs for children, parents and the dyad between pre and post intervention for each costing perspective. Uncertainty around the cost and effect data will be explored using non-parametric bootstrapping and plotted on a cost effectiveness plane [59]. The probability of cost-effectiveness of the intervention at different values of willingness to pay will be estimated and represented visually as a cost-effectiveness acceptability curve [60].

### Patient and public involvement

This project has been co-created together with clinical child health psychologists, a special education teacher, the last author (KF) and the first author (AD). The collaboration between the clinicians and the researchers will continue during the entire project and the clinicians will constitute the Clinical expert group. The researchers and Clinical expert group have monthly meetings. This group has actively participated in the preparation phase of the project, and they are involved in delivering the intervention. Furthermore, they will be invited to interpret the results. However, they will not be collecting data, nor analyzing it, in order to minimize the risk for bias.

In addition to this, we formed Parent Council consisting of four parents of children with subclinical NDDs. The main task of the council was to advise the team on finalizing the design of the project before the ethical application was due in June 2024. They specifically worked on issues of appropriateness and communication with the target group. Examples of specific tasks performed were co-creating advertisement for the project, writing information material and advice on recruitment. They were also asked for feedback on the measurements. Thus, the Parent council has been vital for the project.

### Ethical considerations

The study was granted Ethical approval by the Swedish Ethical Review Authority (2024-03064-01) and will be performed in accordance with the Declaration of Helsinki. Information about the study (aimed at parent and child respectively) will be provided. Informed written consent will be obtained from parents participating in the study. If the child has two guardians, the consent of both is needed for the child to participate in the study. The informed consent includes written information about the study and information about the possibility for participants to withdraw from the study at any time. The template provided by the Swedish Ethical Review Board will be used for the informed consent. Parents or legal guardians of all participating children will give their written informed consent on behalf of their children, prior to inclusion in the study. The children will be asked for their assent before answering the weekly short measures.

Participants will be compensated with vouchers for their involvement in this study. Research publications and data dissemination will not include identifiable data. Swedish General Data Protection Regulation will apply to all aspects of the study.

We have a three-fold communication strategy to disseminate the findings. The first part is aimed at professional community and policy makers on both national and regional levels. The research group and the clinical project group have well established contacts with interested parties both locally, regionally and nationally. We regularly participate and are invited to present at conferences and meetings with municipalities, regions and other bodies.

The second part is aimed at the interested public which can be reached through national patient/interest organizations, social media and traditional media. The third part is to disseminate the findings in the scientific community through publishing the results in suitable scientific journals and presenting them at national and international conferences.

## Discussion

This paper outlines the protocol for the evaluation of the Everyday Life and Parenting group parenting intervention for parents of children aged 2–6 years with subclinical NDDs and mental health problems. Some strengths and limitations of this have been recognized. Firstly, by collecting and evaluating longitudinal data from children and/or their parents, the study will provide valuable insights into the trajectories of mental health and well-being in this group. Secondly, the study will involve pre-school children as informants, providing crucial information about their experiences and perspectives on family life. Thirdly, the use of pre-, post- and follow-up questionnaires will allow reliable and clinically significant changes to be assessed in child emotional and behavioral problems, parental self-efficacy, stress, and behavior.

Despite these strengths, the use of SCED without randomization presents a methodological limitation, as it prevents control for potential confounding variables related to time, such as historical events or maturational effects in children. While this design limitation affects the internal validity of the study, the strengths of this research have the potential to make a significant contribution to understanding the effectiveness of an early parenting support group intervention specifically designed for children with subclinical NDDs. This includes reducing child EBP and parental stress, and improving parental self-efficacy, well-being, and quality of life. The study will also assess the cost-effectiveness of the intervention.

## Data Availability

No datasets were generated or analysed during the current study.

## Abbreviations

ABA: Applied Behavior Analysis
ADHD: Attention Deficit Hyperactivity Disorder
AQOL-8D: Assessing Quality of Life 8 Dimensions
ASD: Autism Spectrum Disorder
CSC: Clinically Significant Change
EBP: Emotional and Behavioral Problems
ECBI: Eyberg Child Behavior Inventory
GHQ-12: General Health Questionnaire
ICER: Incremental Cost-Effectiveness Ratio
ICF: The International Classification of Functioning
MaaP: Me as a Parent
NAP: Nonoverlap of All Pairs
NDD: Neurodevelopmental Disorder
PSS: Parental Stress Scale
QALY: Quality Adjusted Life Years
RCI: Reliable Change Index
REDCap: Research Electronic Data Capture
SCED: Single Case Experimental Design
SDQ: Strengths and Difficulties
TIC-P: Trimbos/iMTA questionnaire for costs associated with Psychiatric Illness
